# Barriers of Adherence and Possible Solutions to Nonadherence to Antidiabetic Therapy in Women with Diabetes in Pregnancy: Patients' Perspective

**DOI:** 10.1155/2017/3578075

**Published:** 2017-07-30

**Authors:** Doreen Mukona, Stephen Peter Munjanja, Mathilda Zvinavashe, Babil Stray-Pederson

**Affiliations:** ^1^Department of Nursing Science, University of Zimbabwe College of Health Sciences, Box A178, Avondale, Harare, Zimbabwe; ^2^Department of Obstetrics and Gynecology, University of Zimbabwe College of Health Sciences, Box A178, Avondale, Harare, Zimbabwe; ^3^Institute of Clinical Medicine, Oslo University and Division of Women and Children, Oslo University Hospital, Rikshospitalet, 00 27 Oslo, Norway

## Abstract

Diabetes in pregnancy contributes to maternal mortality and morbidity though it receives little attention in developing countries. The purpose of the study was to explore the barriers to adherence and possible solutions to nonadherence to antidiabetic therapy in women with diabetes in pregnancy. Antidiabetic therapy referred to diet, physical activity, and medications. Four focus group discussions (FGDs), each with 7 participants, were held at a central hospital in Zimbabwe. Included were women with a diagnosis of diabetes in pregnancy, aged 18 to 49 years, and able to speak Shona or English. Approval was obtained from respective ethical review boards. FGDs followed a semistructured questionnaire. Detailed notes were taken during the interviews which were also being audiotaped. Data were analysed thematically and manually. Themes identified were barriers and possible solutions to nonadherence to therapy. Barriers were poor socioeconomic status, lack of family, peer and community support, effects of pregnancy, complicated therapeutic regimen, pathophysiology of diabetes, cultural and religious beliefs, and poor health care system. Possible solutions were fostering social support, financial support, and improvement of hospital services. Individualised care of women with diabetes is essential, and barriers and possible solutions identified can be utilised to improve care.

## 1. Introduction

Diabetes in pregnancy, both gestational and pregestational diabetes, is a significant clinical and public health problem that has clear relationships with adverse maternal and neonatal outcomes [1]. Gestational diabetes mellitus (GDM) is any degree of glucose intolerance with onset or first recognition during pregnancy [2]. Morbidities of both pregestational diabetes mellitus (PGDM) and GDM in pregnancy include fetal macrosomia, neonatal hypoglycemia, perinatal mortality, polyhydramnios, and increased risk of caesarean delivery [3].

Zimbabwe, with a prevalence of diabetes of 9.7%, is ranked 4th among African countries with the highest prevalence of diabetes mellitus [4]. Poor control of diabetes in pregnancy has been reported in developing countries. Prevalence of poor control in pregnancy of 6%, 20%, 40%, and 58% has been reported in South Africa [5], Sudan [6], Nigeria [7], and Pakistan [8], respectively. Prenatal care is aimed at interventions to promote normoglycemia and reduce maternal and neonatal risks. The intensity of obstetrical and medical management required to optimize glucose control necessitates a patient to learn, commit, and execute self-care tasks and behaviour change for a successful pregnancy [9]. Pregnant women require higher-than-normal levels of engagement with care, adoption of complicated and novel health behaviours, or interaction with new learning systems which can be very complicated [9]. In Zimbabwe, women with diabetes in pregnancy, both GDM and PGDM, are referred for specialized care at tertiary institutions. Risk factor-based screening is done at primary care centres followed by referral to a tertiary care institution if GDM is detected. Some of the risk factors are history of diabetes in a first-degree relative, previous GDM, previous macrosomia, previous unexplained perinatal mortality, and a prepregnancy body mass index above 25 kg/m^2^. The World Health Organization (WHO) criterion for diagnosis is used. Diagnosis of GDM is made if one or more of the following criteria are met: fasting plasma glucose ranging between 5.1 and 6.9 mmol/l (92–125 mg/dl), a 1-hour plasma glucose equal to or more than 10.0 mmol/l (180 mg/dl) following a 75 g oral glucose load, and a 2-hour plasma glucose ranging between 8.5 and 11.0 mmol/l (153–199 mg/dl) following a 75 g oral glucose load. After a diagnosis of GDM, patients are initially admitted for full history taking and physical examination. Assessments include renal function, midstream urine for microculture and sensitivity, fundoscopy, full blood count, weekly cardiotocograph (CTG), and daily fetal movement chart (FMC) from 32 weeks of gestation. Baseline growth parameters are assessed by ultrasound scan that is repeated at 28, 32, 36, and 38 weeks with special emphasis on detection of intrauterine growth retardation (IUGR) and macrosomia. Daily fasting and pre- and postprandial blood glucose monitoring are recommended for all patients. They are put on a thrice-daily soluble insulin regime or a twice-daily regime with a combination of soluble insulin and isophane, if dietary management fails. Patients pay for all these services.

Suboptimal adherence is highly prevalent, is associated with increased morbidity and mortality, is costly to manage, and is the principal obstacle to successful pharmacotherapy in ambulatory patients [10]. Patients have difficulty taking the prescribed medications, following a diet, and changing their lifestyle as directed [11]. Though studies on factors related to nonadherence to therapy in nonpregnant populations with diabetes have been conducted in Zimbabwe, none have been done in pregnant women with diabetes. Adherence to lifestyle modification was low (7.56%) in a study conducted in Zimbabwe, and factors influencing adherence to diet included lack of money (21.01%), attending functions (15.13%), lack of satiety (12.61%), and tempting foods (12.61%) [12]. Factors influencing adherence to exercises were illness (26.89%), lack of time (18.49%), fatigue (15.13%), and no education (10.92%) [12]. The purpose of the study was to explore the barriers to adherence to antidiabetic therapy and the possible solutions to nonadherence in pregnant women with diabetes.

## 2. Methods

The study utilised a descriptive qualitative design, and focus group discussions (FGDs) were conducted. Antidiabetic therapy in this study referred to diet, physical activity, and medications. Four FGDs were held at a central hospital in Zimbabwe, and each had 7 participants who were pregnant at the time of data collection. FGDs were chosen over in-depth interviews because they allow recruitment of relatively more participants in a shorter space of time and allow group interaction thereby yielding more data faster than in in-depth interviews. FGDs each comprised women with PGDM and GDM. The number of focus groups was determined by saturation while the number of participants per FGD was predetermined. The group size enabled all members to talk and share their thoughts while being large enough to create a diverse group. Maximum variation purposive sampling was done for the purpose of documenting diverse variations that are unique that have emerged in adapting to different conditions and identifying important common patterns that cut across variations [13]. Participants who had the highest adherence levels (80% and above) to antidiabetic therapy and those who had the lowest levels (below 50%) were included in the focus group discussions. They were recruited consecutively as they were identified until 7 participants were identified for each group. This helped to identify common barriers and possible solutions to nonadherence to antidiabetic therapy in this population. It also highlighted common experiences in living with diabetes in pregnancy. Included were women with a diagnosis of diabetes in pregnancy, aged 18 to 49 years, and able to speak Shona or English. Approval was obtained from respective ethical review boards. All participants signed an informed consent after full explanation of the nature of the study, its risks, and benefits. FGDs followed a semistructured questionnaire that had sections that had open-ended questions asking about the barriers and possible solutions to adherence to antidiabetic therapy. The researcher moderated and guided the discussion while probing participants for clarity of responses and taking notes. An assistant took detailed notes. All interviews were conducted in a private room, and each lasted about an hour. FGDs were audiotaped and then transcribed verbatim. Trustworthiness was achieved by observing credibility, dependability, transferability, and confirmability. Data analysis commenced in the field during data collection where field notes were being scrutinised for recurring or unusual statements. Data analysis was based on thematic analysis from Miles et al. [14] and Braun and Clarke [15] and it was done manually. The stages of data analysis followed were data organisation, familiarisation, transcription, coding, developing a thematic framework, indexing, displaying, and reporting. Coding was done by the researcher and coresearcher. Discrepancies were critically examined and compared against original transcripts and field notes by both researcher and coresearcher until consensus was reached. Major themes identified were barriers and possible solutions to nonadherence to antidiabetic therapy.

## 3. Findings

### 3.1. Demographics

This section presents the demographic data of the participants.  Table 1 is a summary of all the participants of FGDs (*N* = 28). Twelve (42.86%) had type I diabetes, 10 (53.71%) had type II, and 6 (21.43%) had GDM. Their ages ranged from 19 to 40 years with a mean age of 32.43 years. Twenty-seven (96.43%) were married while 1 (3.57%) was single. Twenty-six (92.86%) participants earned below USD$481 while 2 (7.14%) earned above USD$481. Twenty-three (89.29%) attained ordinary level of education, 4 (14.29%) attained primary level, while 1 (3.57%) attained tertiary level. Twenty-two (78.57%) were unemployed, 4 (14.29%) were self-employed, while 2 (7.14%) were employed.

Table 2 is a diagrammatic representation of the categories identified under the two main themes of barriers and possible solutions.

#### 3.1.1. Poor Socioeconomic Status

The most common cited barrier across all focus groups was lack of finances. In terms of medications, participants reported lack of money to buy medications and glucose testing strips. Though some of them could acquire glucometers, glucose testing strips and syringes were too expensive for them. Participants mentioned difficulty buying the required healthy foods and snacks recommended in diabetes in pregnancy. Regarding exercise, participants generally had no money to join professional and formal gyms or hire personal trainers so they could perform the required physical activity. Though they could jog or brisk walk as forms of exercise that do not require money, they were concerned about their safety and negative reactions of people, especially men, when jogging on the streets. This is expressed in the following excerpts.

*I do not go to work and my husband is not formally employed. He just does casual work which he sometimes fails to find. As a result our family income is not constant and sometimes I fail to buy insulin. Even though I have my glucometer that I got from ZDA, I cannot afford to buy test strips to test myself.* (Age 33, participant 7, FGD 2, type II DM)

*I know the foods that I should be taking with my problem of sugar. Sometimes I do not have money to buy the non-refined foods. They are difficult to find and they are expensive. Beef is even easier to find and cheaper to buy than chicken and fish because one can just buy mincemeat for a dollar.* (Age 40, participant 3 FGD 2, type II DM)

*Exercising in pregnancy is good. The problem is that in our communities you cannot be seen running on the streets with your big tummy. I have tried it before and I was jeered at. I could do it at home but I cannot afford to hire a personal trainer or to buy indoor gadgets that I can use to exercise at home or even to pay monthly for gym.* (Age 35, participant 4, FGD 3, type I DM)

#### 3.1.2. Inadequate Family, Peer, and Community Support

Inadequate family, peer, and community support was expressed across all the focus groups. Women mentioned that they did not get support from their husbands, family members, in-laws, and the communities at large. Some participants mentioned that it was unacceptable in their homes to cook separate meals during meal times as they would be labelled greedy and nagging. It was difficult for most participants to turn down food when they visited or not eat when they attended family functions as this is culturally unacceptable. Some reported that their husbands wished to pamper and spoil them during the pregnancy by buying them fast foods. Refusal to eat the foods provoked resentment from husbands. Some would end up eating wrong foods to maintain peace with family which perpetuated poor glycaemic control.

Because of the high prices of medications and glucose testing strips and the numerous tests and scans that are done during the pregnancy, husbands and in-laws complained that diabetes was expensive and in some instances would place the blame on the pregnant woman. Some participants with PGDM mentioned that they were constantly being compared with other nonpregnant people with diabetes and relatives would not understand the stricter control needed in diabetes in pregnancy. Some participants with GDM mentioned a total lack of support due to lack of knowledge of the family about the new diagnosis of diabetes in pregnancy and the related costs of treatment. This resulted in participants foregoing some of the tests and treatments ordered.

Most participants from high-density suburbs mentioned that jogging on the roads was uncommon in their neighbourhoods and was misconstrued as showing off. The environments are generally unsafe, due to high crime, to come out early in the morning, before it is too hot, to jog. Families also did not understand the need to exercise when one is pregnant. Most husbands were not keen to support their wives in exercise. This really reduced the participants' adherence to physical exercise. In terms of diet, some of the women had this to say:

*My husband buys me Chicken Inn whenever he gets some extra money and when I explain to him that I cannot eat fast foods because of my sugar he accuses me of being ungrateful. Sometimes I do eat to make him happy.* (Age 22, participant 2, FGD 4, type I DM)

*It is very difficult to stick with your diet when you are at home with other people especially in-laws. I need to prepare my own meals that do not have cooking oil and sugar but they will think I am cooking more delicious food that I do not want to give them. Sometimes they will say that I do not want to mix with them or I am greedy. My husband hears all this but he does not say anything. As a result I just eat what has been cooked for everyone to avoid problems.* (Age 31, participant 6, FGD 1, type II DM)

With regard to medications, some of the women had this to say:

*Because of the high price of medications, glucose testing strips and the tests, the family will end up opting for faith healers, traditional healers and use of traditional herbs that are cheaper. I once was taken to the “shrine” for cleansing where I spent 3 days in the bush getting holy water without eating. My husband and in-laws were convinced that I had evil spirits. I collapsed from there and when I was taken to the hospital where I was treated for low bold sugar.* (Age 19, participant 1, FGD 1, type I DM)

Regarding exercise, some of the women had this to say:

*People, especially commuter omnibus conductors jeer at us when we are jogging on the streets. Sometimes there are so many people on the roads who pass nasty comments at us and we get embarrassed. It is very unsafe to come out in the morning when there are few people on the roads because people get attacked by strangers. In the end I just stay at home.* (Age 33, participant 2, FGD 1, GDM)

#### 3.1.3. Complicated Therapeutic Regimen

It was mentioned in all focus groups that the treatment regimen for diabetes in pregnancy was complicated. Pricking oneself daily for insulin was reportedly very painful and uncomfortable. This was particularly so for participants with GDM who had been commenced on insulin. In 3 of the focus group discussions, it emerged that correct procedure of drawing insulin without spilling and contaminating was difficult. Besides being difficult to self-inject, insulin was reportedly difficult to store within its recommended temperatures. Some participants did not have refrigerators at home while those who had them had problems of incessant power cuts. Participants who had comorbidities especially hypertension and those who were taking iron tablets complained of a high pill burden. This further complicated the therapeutic regimen.

*Taking insulin daily is very difficult. I must inject myself twice a day. I find it very difficult to withdraw the correct amount without spilling. Besides the insulin I must take my medications for high blood pressure. Sometimes I forget to take the other medications because they are too many.* (Age 29, participant 5, FGD 1, type I DM)

*Insulin must be stored in a refrigerator. There is no electricity where I stay so I just put my insulin in a cool place. Sometimes it gets discoloured but I continue to use it anyway because I cannot buy another bottle when I have not finished what I am using.* (Age 25, participant 2, FGD 2, type I DM)

#### 3.1.4. Problems of Pregnancy

Women across all focus groups mentioned barriers that are related to pregnancy. These included nausea and vomiting, specific food preferences, lack of appetite, and aversions for certain foods that stopped them from adhering to diet. Some mentioned that reduced intake due to nausea and vomiting resulted in a fall in the blood sugar, and they were hesitant to take insulin at stipulated times. Some mentioned increased appetite and binge eating which is not recommended. Regarding physical activity, women across all groups mentioned tiredness, dizziness, swollen legs, and leg cramps as barriers to adherence.

*With this pregnancy I vomit a lot especially in the morning. I do not feel like eating and the food produces horrible smells. I will spend the whole day without eating anything. I get scared to get my insulin injection because I feel my blood sugar is already too low from not eating. Most of the time I do not have the glucose testing strips to check the sugar to verify.* (Age 31, participant 3, FGD 4, type II DM)

*Even if I would want to run and walk to keep fit, my legs are always swollen and painful. I spend most of my time siting with my legs on a chair like I was advised by the sister at the clinic.* (Age 32, participant 6, FGD 3, type I DM)

#### 3.1.5. Pathophysiology of Diabetes

In some focus groups, participants mentioned barriers related to the pathophysiology of diabetes itself. Frequent hunger (polyphagia) and excessive fluid intake (polydipsia) were barriers to adherence to diet. They ended up eating too much food due to frequent hunger and drank forbidden fruit juices and fizzy drinks as it became monotonous to drink lots of water continuously. This was particularly a problem in GDM participants who had just been diagnosed with diabetes.

*For me it is difficult sometimes with this problem of sugar to eat small frequent meals. I am pregnant too and I eat frequent meals but they will not be small because I will be very hungry. I know it is not allowed with this sugar but I really cannot help it.* (Age 39, participant 6, FGD 4, GDM)

*I get thirsty a lot since I was diagnosed of this sugar. In the first days I used to drink water a lot but nowadays it has become boring, water is tasteless and it is too filling. I alternate water with fruit juices so that I do not get dehydrated.* (Age 27, participant 3, FGD 1, GDM)

#### 3.1.6. Poor Service at the Hospital

Barriers related to poor service at the hospital were reported across all groups. Participants mentioned that when admitted, sometimes there will be no glucose testing strips to check their blood sugar so they know the amounts and types of food to eat. When admitted, the women reported lack of proper foods to eat and the snacks required to maintain normoglycaemia as the hospital also fails to provide them. Participants reported lengthy admissions as a barrier to adherence to diet and physical activity. The lengthy admissions were a result of failure to afford critical tests ordered such as scans and failure to have blood sugar monitored due to lack of glucose testing strips. Participants reported that some tests that were ordered were not available at the hospital but in private where they will be unaffordable to most of them. Some participants mentioned lack of continuity of care because of frequent changes of doctors who sometimes gave conflicting advice. Participants also mentioned long queues in the antenatal clinic when they came for review so they ended up eating foods that are not allowed while in the queue. Participants in some focus groups reported that they were never taught about exercise when they came for review and some only met the dietician when they got diagnosed but never met her again.

*In ANC we are not given special preference like what is done to women with BP. We are made to follow the long queues for hours till we get a chance to see the doctor. I get very hungry in the process and I end up eating corn which is not allowed. The doctors change every time I come and I get to be told a lot of different advice. I cannot even ask questions because of this and as a result I just eat what suits me. Sometimes I do not even come for the review because I fear being caught with high blood sugar and being admitted for a long time.* (Age 35, participant 4, FGD 3, type 1)

*Sticking to diet is even more difficult when admitted in hospital. We do not get the snacks that we must eat in between meals because the kitchen does not have. Even the blood sugar is not tested sometimes unless you have your own machine and strips because the hospital does not have. How then am I supposed to know what to eat?* (Age 33, participant 7, FGD 2, type II DM)

#### 3.1.7. Cultural and Religious Beliefs

Cultural and religious beliefs were mentioned across all the four FGDs. Though participants appreciated the importance of adhering to therapy, sometimes families pressured them into consulting traditional healers and faith healers. Some reported that they would seek help from faith healers because their disease was believed to be linked to demons and generational curses because diabetes runs in families. Consulting traditional and faith healers was cheaper or free in some instances and this appealed to families especially when the patient had no income. Some participants used herbs to control blood sugar as insulin is expensive and difficult to store. There seemed to be interplay between lack of finances and cultural and religious beliefs. This is demonstrated in the following excerpts.

*My in-laws complain that my disease is expensive and is costing their son too much money so they want me to consult a traditional healer as this is much cheaper. They believe the herbs he gives work better than my medication and that they do not have side effects.* (Age 19, participant 1 FGD 1, type I DM)

*My family believes that my illness is a result of evil spirits. Sometimes I am forced to get on fasting programmes with them to cleanse myself of the illness. The last time I tried it I fell very sick and I believe my blood sugar had fallen too low.* (Age 32, participant 6, FGD 2, type II DM)

### 3.2. Possible Solutions to Nonadherence to Antidiabetic Therapy

Three categories were identified under possible solutions to nonadherence to antidiabetic therapy.

#### 3.2.1. Getting Social (Family, Peer, and Community) Support

Social (family, peer, and community) support as a possible solution was mentioned across all the FGDs. Some participants mentioned support groups for women with diabetes in pregnancy. Involvement of husbands in preconceptual and antenatal care to enhance their understanding of diabetes in pregnancy and antidiabetic therapy was mentioned in 3 of the FGDs. Some participants mentioned the importance treatment buddies to foster adherence. Regarding exercise, participants mentioned exercise groups for women with diabetes. It was mentioned across all FGDs that education of patients, husbands, families, communities, and the nation at large would help to garner social support as people got knowledge about diabetes in pregnancy.

*I think if husbands and other family members are enlightened about diabetes in pregnancy they will be more supportive. My husband sometimes thinks that I am exaggerating my illness because he compares me with his mother who also has diabetes. He does not understand the difference between diabetes in someone who is not pregnant and diabetes in a pregnant woman. I am sure if he understands the difference he will be more willing to buy me the required foods, the test strips and also to pay for all the tests that are ordered. Even other people we meet at functions or when we visit will be more receptive of us when they see us being choosy on food.* (Age 36, participant 5, FGD 2, type I DM)

*Family members should support women with sugar. They should be educated on diabetes in pregnancy. It is not a demon but just a disease like any other. If they are taught about it they will not force us to get help from faith healers and traditional healers but will actually encourage us to take our medications and eat the correct foods.* (Age 36, participant 2, FGD 3, type 11)

*I have seen social groups help in people with HIV/AIDS. People meet regularly to share ideas and help each other on any issues regarding their condition, including adherence to therapy. They even follow each other to their homes. I think the same set up can work very well in women with diabetes.* (Age 29, participant 5, FGD 1, type I DM)

#### 3.2.2. Provision of Financial Support, Free Drugs, and Services

Participants across all themes reported that financial support was very important in order to acquire medications, especially insulin, and glucose testing strips. Some mentioned the provision of free laboratory services and scans. Other participants mentioned the establishment of food programmes like what is done for people living with HIV/AIDS. In Zimbabwe, there are food programmes some of which are funded by nongovernmental organisations that are meant for HIV-positive people and their families. Participants across all the FGDs mentioned that the active involvement of the Zimbabwe Diabetic Association (ZDA) will help to ease the shortage of insulin and glucose testing strips.

*One solution that can help us to stick to our medications and diet is to give us free drugs like what is done to people living with HIV/AIDS. Even food programmes should also be established for us so we have access to healthy foods all the time. In South Africa pregnant women get insulin, glucometers and glucose testing strips for free. The numerous blood tests that are ordered should be done for free as well because if I do not know the level of my blood sugar I will just eat anything that comes by. Knowing the level of my blood sugar and the health of my unborn baby helps me to be more responsible on diet and medications.* (Age 34, participant 4, FGD 4, type II DM)

*The ZDA should be more involved in sourcing funds for us so we get the best care. I got the glucometer that I use from ZDA and there are times I would get glucose testing strips from there as well. These days they do not have them. They should continue actively looking for funds to help us.* (Age 19, participant 1, FGD 1, type I DM)

#### 3.2.3. Improvement of Services at the Hospital

It was mentioned across all the FGDs that there is a need to improve services at the hospitals. Improvements mentioned included offering regular and comprehensive care, establishment of a special diabetic care unit for women with diabetes, decentralisation of care to local clinics, involvement of other specialists, and offering of preconceptual care.

*There should be a special diabetic care unit for pregnant women with sugar. This will ensure that we do not spend hours following long queues in ANC, we get health education and there is more personalised care and check-up generally improves because we are relatively fewer than other women without diabetes. We will get to be given comprehensive health education and we understand exactly what we should do to keep our blood sugar low. This will also ensure that we are treated of all our ailments besides sugar such as eyes as this also interferes with my sticking to treatment. Sometimes it is difficult for me to draw the correct amount of insulin because I cannot see properly.* (Age 33, participant 6, FGD 2, type II DM)

*I wish we could be seen at the local clinic because travelling here weekly or two weekly is expensive. Sometimes I miss the dates of appointment and I will never know if I need to change my medications or do anything to lower my blood sugar. It is easier for me to walk to the clinic when I have a problem. I have had my insulin increased twice since I fell pregnant.* (Age 27, participant 7, FGD 1, type I DM)

## 4. Discussion

Diabetes in pregnancy can either be pre-existing type I or type II or gestational diabetes mellitus [16]. Majority of research on diabetes in pregnancy has focused on epidemiological, pathological, and biological aspects of gestational diabetes. Few have focused on the social and behavioural impact from the patients' perspective [17]. A diagnosis of GDM can be associated with anxiety and confusion. Financial, language, and cultural barriers can influence the capacity to comply with health care advice. A total of four focus group discussions each comprising seven women with diabetes in pregnancy were held. The two main themes were barriers and possible solutions to nonadherence to antidiabetic therapy in women with diabetes in pregnancy.

### 4.1. Barriers to Adherence

Seven categories relating to barriers, namely, poor socioeconomic status, lack of family, peer, and community support, effects of pregnancy, pathophysiology of diabetes, complicated therapeutic regimen, religious and cultural factors, and poor service at the hospital, were identified.

Financial barriers were reported across all the four focus group discussions in this study. Participants in this study mentioned lack of finances to buy insulin, glucose testing strips, snacks, and gym equipment. These problems have been highlighted in the literature. Shortages of appropriate test strips have been reported to threaten continuity of care in diabetes in pregnancy [18]. Other authors concur that glucometers are quite affordable but test strips are expensive, device-specific, and not always easy to obtain in developing countries [18]. Participants in this study cited lack of finances to join gyms and hire personal instructors as barriers to physical activity. Collier et al. concur that financial barriers together with difficulties accessing care, barriers to maintaining a healthy diet and exercising, communication difficulties, lack of social support, and barriers related to diabetes care compromise management of diabetes in pregnancy particularly in developing countries [19]. A GDM diagnosis has a financial impact and causes disconnection between diabetes prevention recommendations and specific cultural practices [20]. Participants in this study cited the difficulties associated with the radical changes in financial demands and lifestyle required in GDM as difficult to handle at home. Preventing adverse outcomes depends on one's ability to identify and overcome barriers to proper management of diabetes. However, in this study, majority participants were unemployed and depended on husbands and family for support. This compromised their ability to deal with the demands of diabetes in pregnancy. This has also been reported in the literature by authors who cited nonadherence to therapy related to lack of financial resources and limited access to health care among the most common barriers to care in uninsured women [17]. Majority participants in this study earned less than USD$481 which is the poverty datum line in Zimbabwe.

Lack of family and community support was cited as barriers to adherence to therapy in this study. Lawson and Rajaram reported in their study that women reported lack of reassurance by their health care providers who were not sympathetic and did not offer psychological support [21]. In another study, women complained of difficulty to comply with dietary regimen without receiving adequate nutritional support [22]. Cultural and religious beliefs were cited in this study as barriers to adherence to therapy. It is important to pay attention to specific cultural issues of diabetic pregnant women in order to help them with effective self-management skills.

Problems of pregnancy, nausea and vomiting, lack of appetite, cravings, and aversion for certain foods were also cited as barriers to adherence to diet. Women reported that they sometimes had aversion to certain healthy foods. Some complained that water is tasteless and difficult to have as the only fluid. Other factors mentioned were dizziness, tiredness, and swelling of limbs. Physical restrictions to exercise in pregnancy were also reported in an Australian study on women with GDM [23]. Clinical features of diabetes such as polyuria, polydipsia, polyphagia, and fatigue were also mentioned, and these together with the effects of pregnancy further reduce adherence to therapy [24].

Participants in this study reported a complicated therapeutic regimen that they found overwhelming to follow. They cited the complexity of storage of insulin, self-injection, and self-monitoring of blood glucose on a daily basis. Provision of basal and prandial insulin needs with intensified insulin regimens is necessary for optimal glycaemic control. Prandial insulin needs to be matched to carbohydrate intake, premeal blood glucose, and anticipated activity [25]. All this can be complicated and overwhelming to a woman with diabetes in pregnancy. This is consistent with low self-efficacy and that has been reported in the literature [9, 26]. Women in other studies have reported being overwhelmed by the quantity and complexity of unfamiliar nutrition recommendations [9]. Self-efficacy promotes diabetes self-care [27]. Findings of a qualitative study conducted in China revealed that women felt confusion, anxiety, and guilt about GDM. They found the dietary requirements confusing, felt hungry all the time, were not aware of appropriate food substitutions, and thought they could transmit the diabetes to their babies through breastfeeding. They generally felt information from friends, magazines, a health phone line, and the Internet was insufficient, and they needed to learn from small group sessions and information leaflets [28].

In this study, women cited lack of sufficient time for consultation with health care professionals. It has been reported in the literature that there is a critical shortage of health care workers in low- and medium-income countries that is compounded by prioritisation of such conditions as HIV/AIDS and malaria [29]. Other health system barriers reported in the literature are lack of trained staff, standard protocols, consumables, financing of services and treatment, and referral systems [29]. However, health services and systems in low- and middle-income countries are disorganised and inadequately financed and can work as barriers for achieving specific health-related outcomes [29]. Referral to a higher level of care for a woman with diabetes in pregnancy results in additional costs and may result in nonadherence to therapy [29]. Many developing countries do not have specialists for diabetes care [30]. This can limit access to specialized services and causes treatment delays [31]. Referral for a condition that in 70–85% of cases can be successfully treated with diet only is a waste of resources, time, and money [32]. However, initiating management that starts with nutritional therapy through the local health centre would require additional training for primary health care workers [31].

This underscores the importance of intensive and individualised health education of patients to foster adherence to therapy in diabetes in pregnancy. However, lack of knowledge has also been reported in the literature as one of the problems of management of type II diabetes in low- and middle-income countries [33]. Lack of information about GDM and barriers to receiving and accessing health care have been reported as the major concerns amongst low socioeconomic status women with GDM [19].

### 4.2. Possible Solutions to Nonadherence

The possible solutions identified in this study can be used to improve care of women with diabetes in pregnancy. Care during pregnancy in women with diabetes can be improved by understanding factors that promote diabetes self-efficacy or confidence in performing diabetes-related health behaviours [27]. Categories identified under possible solutions were provision of free health care and services, provision of family and community support, and improvement of services at the hospital.

A supportive social and physical environment has been identified as a promoter of diabetes self-care among low-income women [27]. Social support provides a helpful motivation for maintaining normoglycemia [19]. Some studies identified support from clinical office staff as a facilitator of postpartum follow-up care after gestational diabetes [34].

Improvement of services at the hospital was cited as a facilitator of adherence to therapy in this study. Improvement of service delivery will also address a number of financial barriers imposed by poor socioeconomic status. Women in this study mentioned availability of trained staff, establishment of a unit dedicated to the care of women with diabetes, availability of drugs and consumables, availability of proper diabetic diet, and more time with health care professionals during consultation for asking questions and voicing concerns related to diabetes in pregnancy. They also cited the importance of the physiotherapist in their care to give them health education on proper exercise during pregnancy. Screening for gestational diabetes and management of uncomplicated cases through the primary health care level could ease access to care and facilitate the surveillance of affected women within the continuum of care. Strengthening the role of the primary level of care in GDM management could contribute to the prevention of future diabetes in affected mothers and their children [35].

To ease the problems of nonadherence, dietary requirements must be translated and adapted to local eating patterns and product availability. There is a need to consider cheaper medications than insulin especially in GDM and pregestational type II diabetes. Metformin is not yet recommended as an oral alternative for insulin despite its proven comparability [36], but its ease of use, and reduced risk of hypoglycaemia, less strict glucose monitoring, lower cost, and having no particular storage and refrigeration requirements makes it a better and safer alternative for developing countries.

Training of health care providers in the area of diabetes in pregnancy needs to be initiated or scaled up to ensure that women with diabetes in pregnancy are properly managed, including proper health education. Women diagnosed with gestational diabetes are usually referred to a specialist despite general practitioners at health centre level being responsible for the management of nonpregnant diabetic patients. Decentralization of screening for gestational diabetes and initial management of uncomplicated cases at the primary level of care could ease access to care and reduce the number of mothers who are diagnosed after a complication occurred [35].

Although regular visits to a specialist care team are important, in settings where access to health care is a major obstacle, alternative solutions need to be developed. Phone consultations could help improve access to care, although these would require autonomous patients who are able to perform self-monitoring of blood glucose (SMBG) [37]. SMBG also requires women to be able to read and write, which is a challenge in many sub-Saharan African and South Asian countries with high rates of female illiteracy [38]. Home visits by community health agents [39] or community-based diabetes groups could improve monitoring of women with diabetes in pregnancy.

This study has its limitations. The FGDs were done with a purposive sample of pregnant women with diabetes in pregnancy, and maximum variation sampling was employed. The use of self-reported adherence could have introduced bias as self-reports tend to overestimate adherence. Purposive sampling done limits the generalizability of the findings to the entire population of women with diabetes in pregnancy. However, the study generated important insights into the challenges that pregnant diabetics face in self-management. These can be utilised to develop management protocols that are both relevant to the target population and culturally acceptable in the setting.

## 5. Conclusion

In conclusion, participants in this study had challenges in adherence to diet, physical activity, and medications. Financial barriers and lack of support were mentioned across all focus groups. They however highlighted possible solutions to the challenges identified. There is a need to develop culture-specific protocols in the management of diabetes in pregnancy.

### 5.1. Implications to Practice

Pregnant women with diabetes face a lot of barriers in adhering to antidiabetic therapy. Health care workers need to be trained in the management of diabetes in pregnancy in order to reduce adverse perinatal outcomes. There is need to include culture-specific health education and interventions to improve adherence to antidiabetic therapy in order to reduce the incidence of adverse perinatal outcomes. Husbands and families should be actively involved in the management of diabetes in pregnancy so they embrace the demands imposed by diabetes in pregnancy. Decentralisation of care to primary health care centres can help improve access to care. Cheaper alternatives to insulin therapy such as metformin can be used in the management of type II diabetes and GDM. There is a need to simplify diabetic protocols especially in GDM by using oral hypoglycaemic agents which are much cheaper than insulin. Rather than jogging as exercise, women can be advised to brisk walk especially after meals to control post prandial hyperglycaemia. Women can also be trained to exercise in the comfort of their own homes. Peer educators can be trained to give health education and support to families affected by diabetes in pregnancy. However, though important insights were generated out of the study, generalisability to the general population might be limited by the qualitative nature of the design.

## Figures and Tables

**Table 1:** Summary of demographic characteristics for participants for FGDs (*N* = 28).

Variable	Frequency	Percentage
Age in years		
19–25	4	14.29
26–30	4	14.29
31–35	13	46.43
39-40	7	25.00
Type of diabetes		
Type 1	12	42.86
Type 2	10	35.71
GDM	6	21.43
Marital status		
Married	27	96.43
Single	1	3.57
Employment status		
Employed	2	7.14
Unemployed	22	78.57
Self employed	4	14.29
Monthly income		
<$481	26	92.86
>$481	2	7.14
Level of education		
Primary level	4	14.29
Ordinary level	23	82.14
Tertiary level	1	3.57

**Table 2:** Summary of categories identified under barriers and possible solutions to nonadherence to antidiabetic therapy.

Theme	Categories
Barriers of adherence	(1) Poor socioeconomic status (2) Lack of family, peer, and community support (3) Effects of pregnancy (4) Complicated therapeutic regimen (5) Pathophysiology of diabetes mellitus (6) Cultural and religious beliefs (7) Poor health care system

Possible solutions to nonadherence	(1) Fostering family, peer, and community support (2) Getting financial support (3) Improvement of service at the hospital
